# Synthesis, Characterization and *in Vitro* Evaluation of New Composite Bisphosphonate Delivery Systems

**DOI:** 10.3390/ijms150916831

**Published:** 2014-09-22

**Authors:** Joanna Kolmas, Marcin Sobczak, Ewa Olędzka, Grzegorz Nałęcz-Jawecki, Cezary Dębek

**Affiliations:** 1 Department of Inorganic and Analytical Chemistry, Faculty of Pharmacy, Medical University of Warsaw, ul. Banacha 1, 02-097 Warsaw, Poland; E-Mails: joanna.kolmas@wp.pl (J.K.); eoledzka@wp.pl (E.O.); 2 Department of Environmental Health Science, Faculty of Pharmacy, Medical University of Warsaw, ul. Banacha 1, 02-097 Warsaw, Poland; E-Mail: gnalecz@wum.edu.pl; 3 Institute for Engineering of Polymer Materials and Dies, ul. Marii Skłodowskiej-Curie 55, 87-100 Toruń, Poland; E-Mail: c.debek@ipgum.pl

**Keywords:** polymeric biomaterials, bisphosphonates, polyurethanes, hydroxyapatite, drug delivery systems, clodronate

## Abstract

In this study, new composite bisphosphonate delivery systems were obtained from polyurethanes (PUs) and nanocrystalline hydroxyapatite (HA). The biodegradable PUs were first synthesized from poly(ε-caprolactone) diols (PCL diols), poly(ethylene adipate) diol, 1,6-hexamethylene diisocyanate, 1,4-butanediol and HA. Moreover, the PCL diols were synthesized by the ring-opening polymerization catalysed by the lipase from *Candida antarctica*. Next, composite drug delivery systems for clodronate were prepared. The mechanical properties of the obtained biomaterials were determined. The cytotoxicity of the synthesized polymers was tested. The preliminary results show that the obtained composites are perspective biomaterials and they can be potentially applied in the technology of implantation drug delivery systems.

## 1. Introduction

Bone metastasis is prevalent in many cancers, especially breast, prostate or lung cancer, the most common neoplasms in the world today [1,2]. Cancer patients with bone metastasis are exposed to numerous skeletal disorders, such as unexpected pathological fractures, serious hypercalcaemia or severe bone pain which is difficult to relieve [1]. Until now, the first-line treatment for bone metastasis has administered bisphosphonates (BPs) [1,2,3,4]. Their mechanism of action is now more clear [5,6,7]. It is commonly known that they inhibit bone resorption by suppressing osteoclastogenesis and osteoclast activity via the farnesyl pyrophosphate synthase enzyme (FPPS) in the mevalonic acid pathway [6,7]. Moreover, recent research shows that BPs may inhibit bone tumour growth and tumour cell invasion in the extracellular matrix. These studies suggest a preventive role played by BPs in tumour metastasis in bone tissues [8,9].

Among the most effective BPs in bone metastasis treatment are: clodronate (CLO), pamidronate, ibandronate and zoledronic acid [2,5,10]. They are administered to patients via two routes—oral or intravenous—though unfortunately they can cause some side effects, such as an acute systemic inflammatory reaction, ocular inflammation, nephrotic syndrome or electrolyte imbalance [11]. Moreover, when applied orally, the bioavailability of these drugs is very low and often insufficient. Thus, it seems reasonable to deliver BPs locally and as a consequence accelerate their local bioavailability.

It should be emphasized that the therapeutic efficacy of BPs administered by standard methods is also limited. In recent years, unconventional macromolecular drug delivery systems (DDS) have became the focus of interest [12,13,14]. Polymeric DDS exhibit unique pharmacokinetics, distribution and pharmacological efficacy. Numerous BP delivery systems (BPDDS) have been investigated [15,16,17,18,19,20,21,22,23]. They include dendrimeric polymers, hydrogels, liposomes, nanocapsules, nanospheres and macromolecular conjugates. One particularly interesting kind of BPDDS comprises biomaterials used as orthopaedic implants [15,16].

Aliphatic or cycloaliphatic polyurethanes (PUs) demonstrate good biodegradability and biocompatibility in human tissues. These attributes make them advantageous and extremely useful for the technology of controlled DDS [14,24].

Until now, the studies into BPDDS have been carried out mostly using animal models, with only a few exceptions employing human clinical trials. As such, the preparation of novel BPDDS is especially interesting for the pharmaceutical industry and medicine in general.

Moreover, it is important to know that BPs exhibit a strong affinity to nanocrystalline hydroxyapatites [25,26]. It has been shown that they may strongly adsorb on the apatitic surface by an ion-exchange mechanism between phosphonate groups from BPs and phosphate ions from hydroxyapatite (HA) [27]. Several authors have reported that some BPs may also adsorb on HA through surface binding of phosphonate groups and Ca^2+^ sites of HA [25,28]. Therefore, the use of HA nanoparticles as the delivery system of BPs has been widely studied [29,30].

The main aim of our study has been to prepare composite CLO DDS using biodegradable PUs hydroxyapatite (HA) as components. The structures and chemical compositions of the new biomaterials were investigated and discussed based on the results obtained.

## 2. Results and Discussion

### 2.1. Synthesis of Polyols and Polyurethanes

The aim of the first part of this study was to obtain poly(ε-caprolactone) diols (PCL diols) which could be applied as precursor of further polyurethanes (PUs) synthesis. The polymerization reactions of ε-caprolactone (CL) in the presence of diethylene glycol (DEG) and the lipase from *Candida antarctica* (CA) were conducted at 70 °C for 14 days. The molar ratio of CL/DEG was either 20:1 (**PCL-1**), 30:1 (**PCL-2**) or 40:1 (**PCL-3**). Reactions were carried out with one level of lipase concentration at the same scale of monomer (100 mg of CA).

The structure of the obtained PCL diols was confirmed by proton nuclear magnetic resonance (^1^H NMR) or carbon-13 nuclear magnetic resonance (^13^C NMR), Fourier transform infrared spectroscopy (FTIR) and matrix-assisted laser desorption/ionization mass spectrometry (MALDI-TOF MS) (Experimental Section).

**Figure 1:**
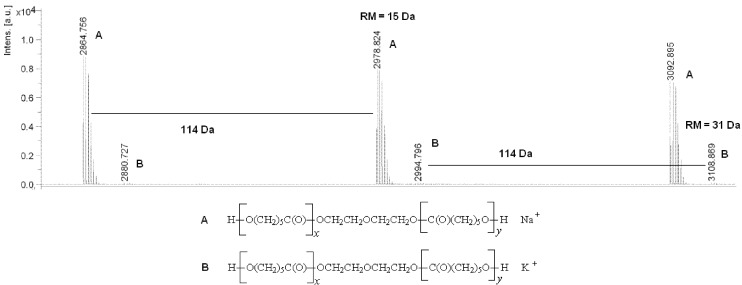
Matrix-assisted laser desorption/ionization mass spectrometry (MALDI-TOF MS) spectrum of poly(ε-caprolactone) diol.

In the MALDI-TOF MS spectra of the PCL diols, linear macromolecules were observed (Figure 1). The first and most prominent series of peaks was assigned to polyols terminated with a hydroxyl group (residual mass: 15 Da, Na^+^ adduct). This series of peaks was differing by 114 Da, which is equal to the mass of the repeating unit of PCL. In addition, low-intensity series of peaks corresponding to macromolecules (residual mass: 31 Da, K^+^ adduct) was detected in the mass spectrum. The average molecular mass (*M_n_*) values of PCL diols determined by the MALDI-TOF MS method ranged from 1500 to 2900 Da (polydispersity indexes (*PD*) 1.36–1.89).

The weight method was used to determine the reaction yield. For the **PCL-1**, **PCL-2** and **PCL-3**, the corresponding yield values were 84%, 73% and 69%, respectively.

The *M_n_* values of the PCL diols determined by the gel permeation chromatography (GPC) method were 1800 (**PCL-1**), 2400 (**PCL-2**) and 3200 Da (**PCL-3**), respectively, while the *PD* showed small variations (between 1.42 and 1.63).

The PUs were obtained using PCL diols, dihydroxy(polyethylene adipate) (PEAD) as the soft segments, and 1,6-hexamethylene diisocyanate (HMDI) and 1,4-butanediol (BD) as components of the hard segments. 1,4-diazabicyclo[2.2.2]octane (DABCO) was used as the polyaddition catalyst. A two-step melt polymerization procedure was engaged to this process. The isocyanate index (isocyanate to hydroxyl equivalent ratio) was 1.05. The molar ratio of HMDI/BD/PEAD/PCL/DABCO was 2.5:0.9:0.8:0.8:0.01. The *M_v_* values of the PUs were evaluated by the viscosity method and were within the range of 58,000–62,000 g/mol (Table 1).

**Table 1:** Characterization of synthesized polyurethanes.

No.	PU	Reagents	*M_v_* (g/mol)	*F_S_* (MPa)	*S_100_* (MPa)	ε (%)	*Sh_H_* (Shore A)
1.	**PU-1**	HMDI/BD/PEAD/PCL-1	62,100	14.7 ± 0.8	5.2 ± 0.3	312 ± 12	44 ± 3
2.	**PU-2**	HMDI/BD/PEAD/PCL-2	58,200	14.3 ± 0.9	5.4 ± 0.3	348 ± 13	42 ± 3
3.	**PU-3**	HMDI/BD/PEAD/PCL-3	59,600	13.9 ± 0.7	5.5 ± 0.2	358 ± 11	41 ± 2

*M_v_—*calculated from the Mark–Houwink equation using the following constants: K = 6.80 × 10^−5^ dL/g and α = 0.86; *F_S_*—fail stress; *S_100_*—stress at 100% elongation; ε—elongation at break; *Sh_H_*—Shore hardness.

**Figure 2:**
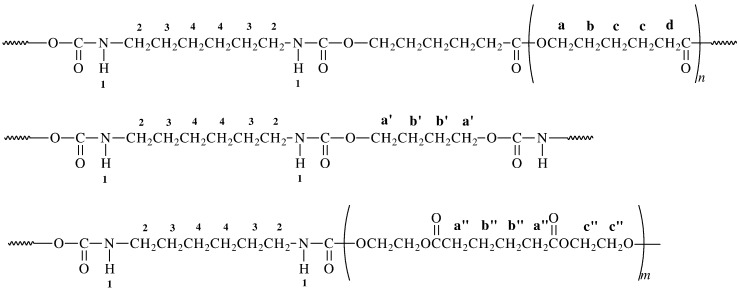
Structure of the obtained polyurethanes.

**Figure 3:**
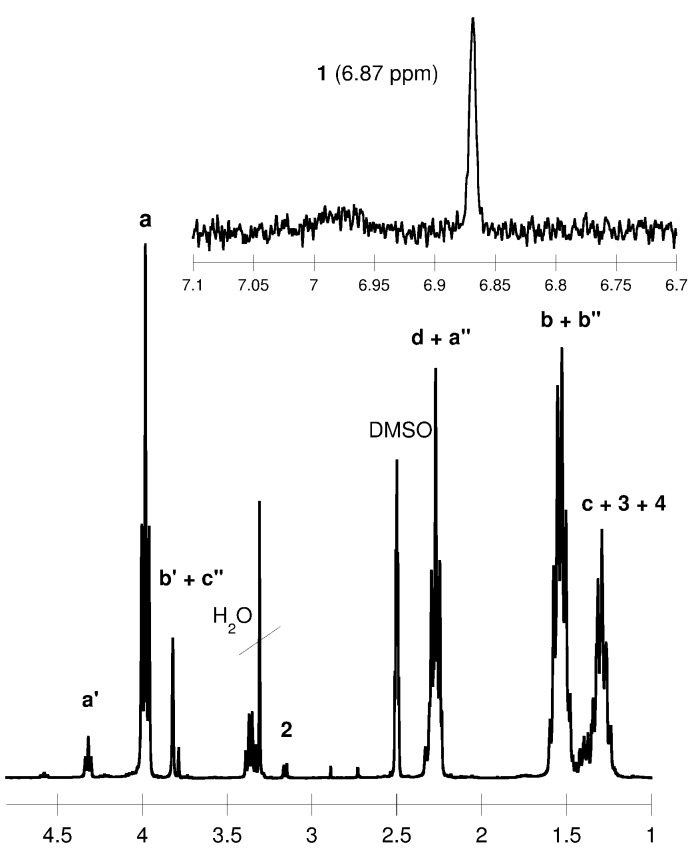
^1^H NMR spectrum of the obtained polyurethane (DMSO-d_6_).

The chemical structure of the PUs was confirmed by ^1^H, ^13^C NMR and FTIR (Figure 2 and Figure 3). The data are shown in the Experimental Section.

Table 1 shows the mechanical properties of the obtained PUs. The fail stress (*F_S_*), stress at 100% elongation (*S_100_*), Shore hardness (*Sh_H_*) and stress at 100% elongation at break (ε) of the obtained PUs were determined. As is presented in Table 1, the ε was greater than 300%. The obtained materials showed *F_S_* within the range of 13.9–14.7 MPa and *Sh_H_* within the range of 41–44 Shore A degrees.

### 2.2. The Polyurethane/Hydroxyapatite Composites’ Fabrication

**Figure 4:**
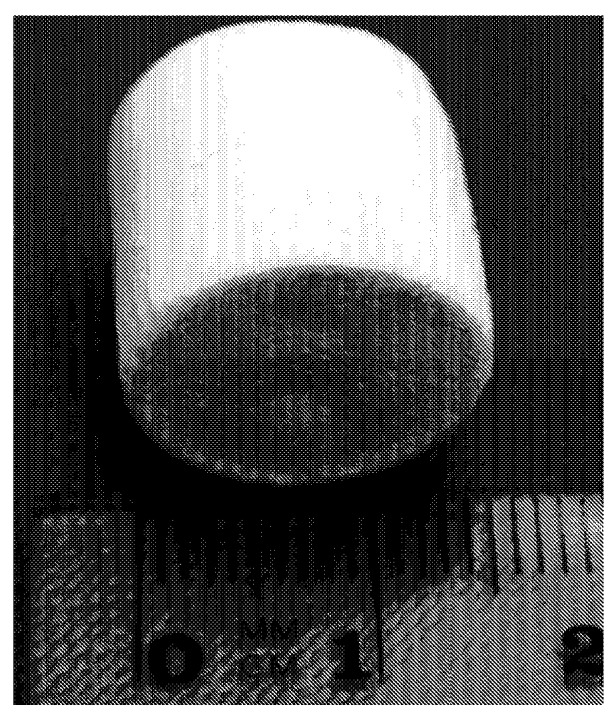
Representative image of the polyurethane/hydroxyapatite composite.

**Figure 5:**
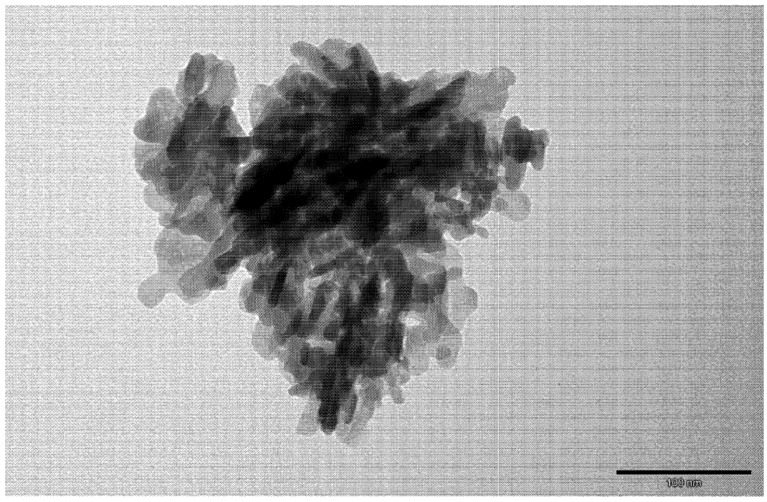
Transmission electron microscope (TEM) image of the hydroxyapatite used as components of the composites.

The polyurethane/hydroxyapatite composites (PU-HA composites) were obtained from previously synthesized **PU-1**, **PU-2** and **PU-3** (Table 2). The ratio of PU to HA was 9:1 or 8:2 (*w*/*w*) (Figure 4). The composites were formed by mixing the mixture of PU and nanocrystalline HA with NaCl. The size of the HA crystals used varied from 15 to 40 nm (Figure 5).

**Table 2:** Characterization of polyurethane/hydroxyapatite composites.

No.	Code	Composition	*d* (g/cm^3^)	*P* (%)
1.	**PU-HA-1**	PU-1(PCL-1)/HA: 9/1	0.225 ± 0.003	66.5 ± 0.9
2.	**PU-HA-2**	PU-1(PCL-1)/HA: 8/2	0.266 ± 0.003	53.6 ± 0.6
3.	**PU-HA-3**	PU-2(PCL-2)/HA: 9/1	0.210 ± 0.002	59.4 ± 0.6
4.	**PU-HA-4**	PU-2/HA PU(PCL-2): 8/2	0.259 ± 0.002	46.4 ± 0.4
5.	**PU-HA-5**	PU-3(PCL-3)/HA: 9/1	0.207 ± 0.002	56.2 ± 0.5
6.	**PU-HA-6**	PU-3(PCL-3)/HA: 8/2	0.244 ± 0.003	42.2 ± 0.5

*d—*the density of the composite; *P—*amount of open pores in the composite.

The density (*d*) increased with HA content. The *d*values were ranged from 0.207 to 0.266 g/cm^3^. In parallel, the porosity (*P*) of the PU-HA composites decreased when HA content increased. For example, the *P* of **PU-HA-1** and **PU-HA-2** was 66.5% and 53.6%, respectively.

**Figure 6:**
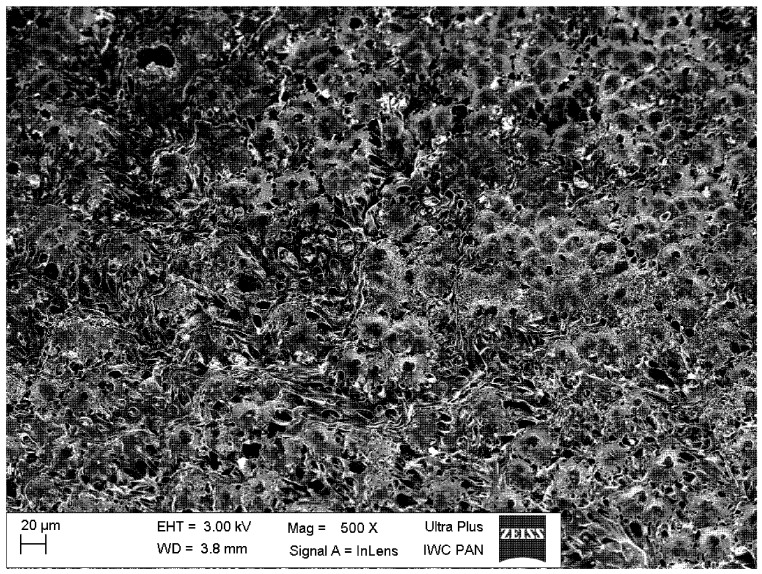
Scanning electron microscope (SEM) micrograph of the polyurethane/hydroxyapatite composite.

A typical scanning electron microscope (SEM) micrograph of the PU-HA composite shows the continuous structure of interconnected and somewhat regular pores (Figure 6). The regular pores range from several microns up to a dozen or so microns, which is within the appropriate range for tissue engineering.

### 2.3. Cytotoxic Tests

Cytotoxic tests of the obtained PUs were carried using the luminescent bacteria *V. fischeri* and two ciliated protozoa *S. ambiguum* and *T. termophila* (Table 3). All the tested samples were not toxic to any of the tested bionts, whether bacteria or protozoa, due to the fact that a sample is considered toxic when the percentage of the toxicity effect (*PE*) is higher than 20.

**Table 3:** Cytotoxicity of the obtained polyurethanes.

PU	Spirotox (24 h)		Microtox (15 min)		Protoxkit F (24 h)	
Concentration (mg·mL^−1^)	10	1	1	0.5	1	0.5
**PU-1**	0	0	0	0	15	5
**PU-2**	0	0	0	0	14	8
**PU-3**	0	0	0	0	16	11

### 2.4. Drug Release from the Polyurethane/Hydroxyapatite Composites

CLO was incorporated into the PU-HA composites by immersing the material in an aqueous drug solution of a known concentration. The drug content in the PU-HA composites was 1% wt.

**Figure 7:**
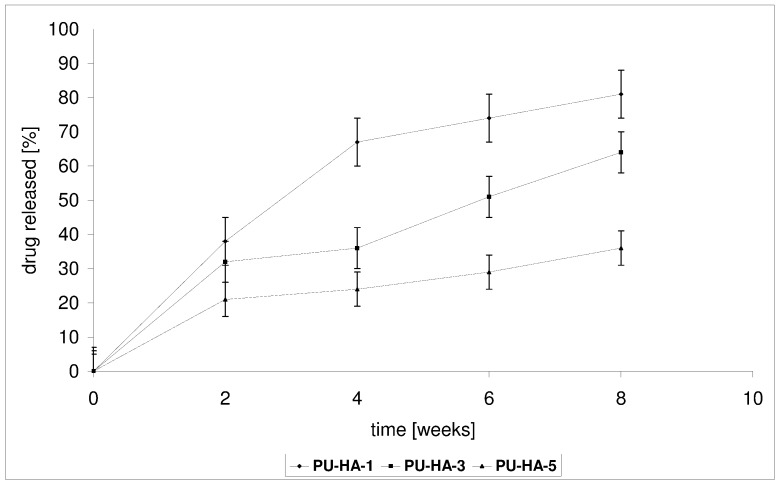
Release of CLO from the PU-HA-1, PU-HA-3 and PU-HA-5 composites.

**Figure 8:**
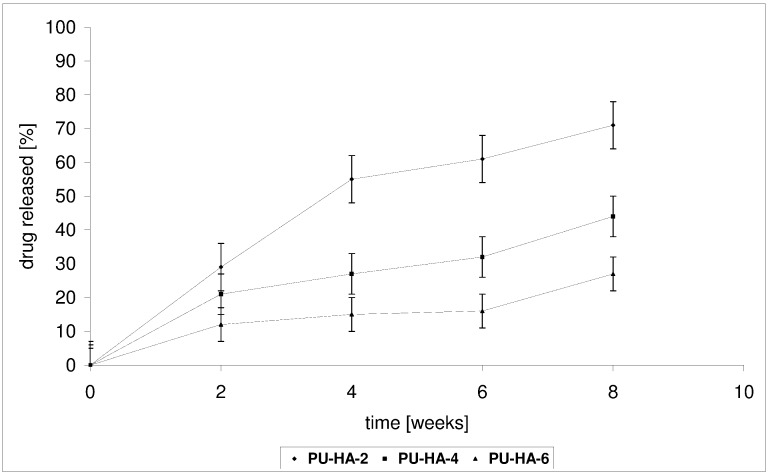
Release of CLO from the PU-HA-2, PU-HA-4 and PU-HA-6 composites.

*In vitro* CLO release from the PU-HA composites was conducted in a phosphate buffer solution (PBS) buffer at 37 °C for 1–8 weeks. The kinetic rates of CLO released from the obtained biomaterials at pH 7.4 are shown in Figure 7 and Figure 8.

Two factors could influence the release of CLO from the obtained PU-HA composites, namely the *M_n_* of the PCL diols used in PU synthesis and the *P* of the composites.

**PU-HA-1** and **PU-HA-2** released CLO faster compared to **PU-HA-3** and **PU-HA-4** or **PU-HA-5** and **PU-HA-6** (Figure 7 and Figure 8). The percentage of CLO released was about 81% for **PU-HA-1**, 64% for **PU-HA-3** and 36% for **PU-HA-5** after eight weeks of incubation. In comparison, the percentage of CLO released after eight weeks of incubation was around 71% for **PU-HA-2**, 44% for **PU-HA-4** and 27% for **PU-HA-6**.

It was found that the rate of CLO release increases with increasing the *P* and decreasing the *M_n_* of PCL used in PU synthesis. The *P* of the PU-HA composites decreases with increasing HA content.

It was already known that the hydrolytic stability of PU-HA composites and CLO release from matrices depend on numerous factors, such as composition, kind of hard or soft segments, the crystallinity, and the size and form of the crystallite of the PU, *etc.* However, it seems that in our study the main influence on this property has a kind of polyols type soft segments.

The degradation tests of the obtained PUs or PU-HA composites and the kinetic rates of the CLO released were conducted in the same manner and under the same conditions.

**Figure 9:**
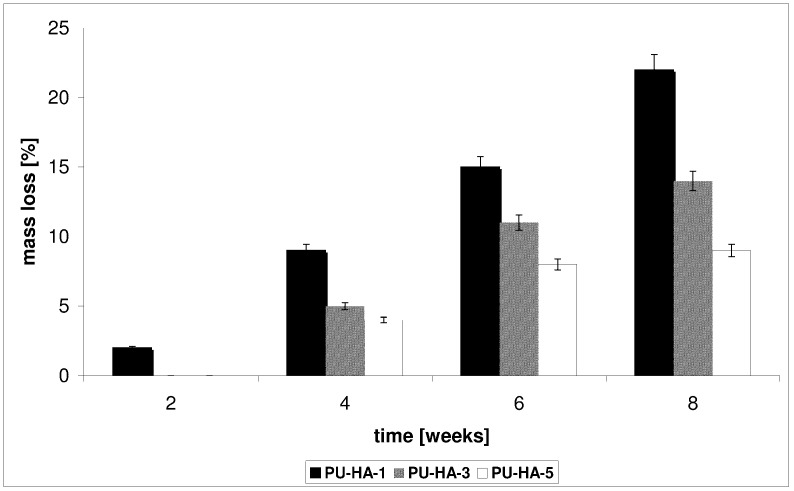
Mass loss of the PU-HA-1, PU-HA-3 and PU-HA-5 composites after the biodegradation process.

**Figure 10:**
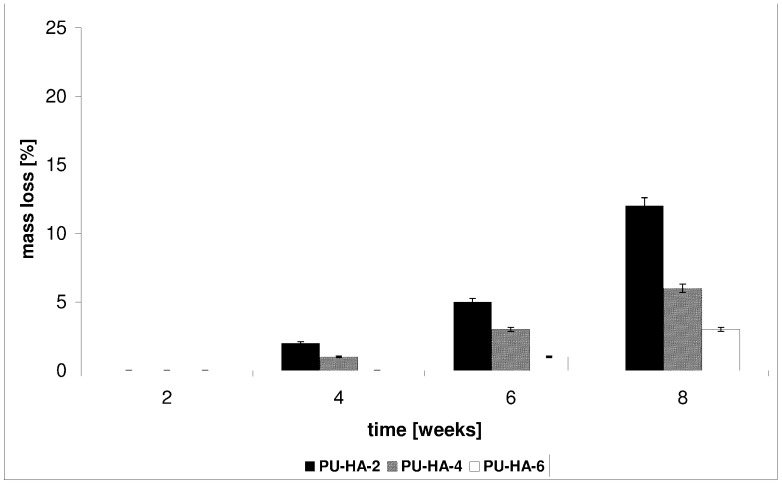
Mass loss of the PU-HA-2, PU-HA-4 and PU-HA-6 composites after the biodegradation process.

The results directly comparing CLO release with the *M_v_* of the PUs (Table 4) or the mass loss (*WL*) of the PU-HA matrices studies follow the same trend (Figure 9 and Figure 10).

*In vitro* degradation of the synthesized PUs was controlled by the change of the mechanical properties and the *M_v_*. The *M_v_* of the obtained PUs were determined after four and eight weeks of degradation (Table 4). The changes in the *M_v_* for the obtained PUs were around 4.7%–6.0% after four weeks and 7.2%–11.4% after eight weeks. **PU-1** degraded faster in comparison to **PU-2** and **PU-3**. The above parameters are in a good agreement with the results of the kinetic rates of CLO release from the obtained PU-HA composites.

**Table 4:** Molecular weight-change of the obtained polyurethanes during the *in vitro* degradation process.

No.	PU	Reagents	*M_v_* (g/mol)	*M_v(4)_* (g/mol)	*M_v(4) loss_* (%)	*M_v(8)_* (g/mol)	*M_v(8) loss_* (%)
1	**PU-1**	HMDI/BD/PEAD/PCL-1	62,100	58,400	6.0	55,000	11.4
2	**PU-2**	HMDI/BD/PEAD/PCL-2	58,200	55,200	5.2	53,300	8.4
3	**PU-3**	HMDI/BD/PEAD/PCL-3	59,600	56,800	4.7	55,300	7.2

*M_v_—*calculated from the Mark-Houwink equation using the following constants: K = 6.80 × 10^−5^ dL/g and α = 0.86; *M_v(4)_*—after degradation (4 weeks); *M_v(8)_*—after degradation (8 weeks).

Furthermore, the kinetic rates of CLO release are in agreement with the change in the mechanical properties of the PUs and the *in vitro*degradation of the produced PU-HA composites. After eight weeks’ degradation process, the **PU-1** obtained from **PCL-1** retained around 76% of the original value of *F_S_*, 84% of the value of *Sh_H_* and 81% of the value of ε (Table 1 and Table 5). The changes of these parameters were clearly smaller for the PUs obtained from **PCL-2** and **PCL-3**, which confirms earlier reports of the higher hydrolytic stability of PUs containing longer polyester units. **PU-3** retained around 88% of the original value of *F_S_*, 93% of the value of *Sh_H_* and 91% of the value of ε (Table 5).

**Table 5:** Properties of the synthesized polyurethanes after the biodegradation process.

No.	PU	Reagents	*F_S_* (MPa)	*S_100_* (MPa)	ε (%)	*Sh_H_* (Shore A)
1.	**PU-1**	HDI/BD/OEAD/PCL-1	11.2 ± 0.7	4.1 ± 0.2	252 ± 10	37 ± 3
2.	**PU-2**	HDI/BD/OEAD/PCL-2	11.7 ± 0.8	4.5 ± 0.3	296 ± 9	37 ± 3
3.	**PU-3**	HDI/BD/OEAD/PCL-3	12.2 ± 0.6	5.0 ± 0.2	326 ± 11	38 ± 2

*F_S_—*fail stress; *S_100_*—stress at 100% elongation; ε—elongation at break; *Sh_H_*—Shore hardness.

*In vitro* degradation of the obtained PU-HA composites was controlled by the *WL* of the materials. The results are shown in Figure 9 and Figure 10. The *WL* values of **PU-HA-1** and **PU-HA-2** were 22% and 12% after eight weeks of degradation, respectively. However, for **PU-HA-5** and **PU-HA-6** the *WL* was 9% and 3% (after eight weeks). The *WL* values of PU-HA increased slowly with increasing hydrolytic degradation time. These results correlate well with the change in the mechanical properties of the PUs.

**Figure 11:**
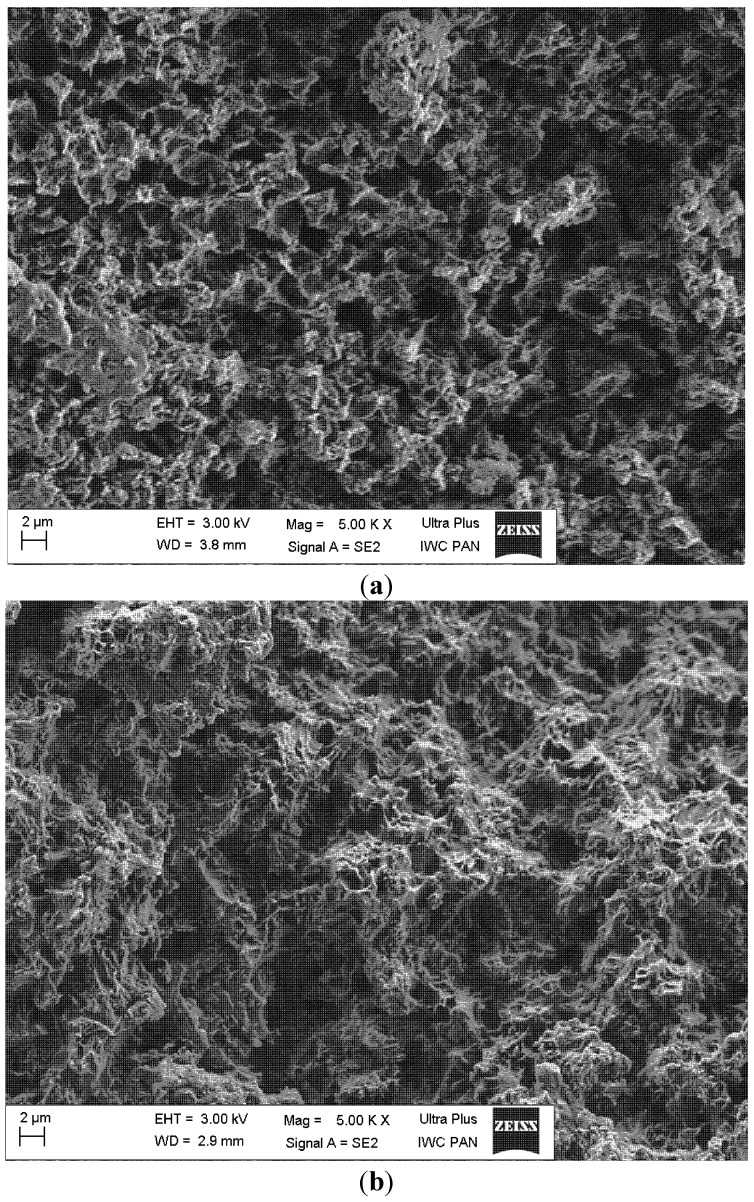
SEM micrographs of the polyurethane/hydroxyapatite composite.(**a**) before the degradation process; (**b**) after eight weeks’ degradation process.

The scanning electron microscopic images of the PU-HA composites, both in their original state and after eight weeks’ degradation process, are shown in Figure 11. In comparison to the original composite (Figure 11a), the surface of the PU-HA composite after the degradation process shows severe cracking all over its surface, indicating significant oxidative/hydrolytic damage (Figure 11b).

## 3. Experimental Section

### 3.1. Materials

Lipase from *Candida Antarctica* (CA, Aldrich, Poznań, Poland), dichloromethane (CH_2_Cl_2_, Polish Chemical Reagents, Gliwice, Poland), dimethyl sulfoxide (DMSO, 99%, Aldrich), *N*,*N*-dimethylformamide (DMF, Aldrich), 2-[(4-hydroxyphenyl)diazenyl]benzoic acid (HABA) and methanol (MeOH, Polish Chemical Reagents) were used as received. 1,6-hexamethylene diisocyanate (HMDI, 98%, Aldrich), poly(ethylene adipate) diol (PEAD diol, *M_n_* = 1000 Da, Aldrich), 1,4-butanediol (BD, 98%, Fluka, Poznań, Poland), 1,4-diazabicyclo[2.2.2]octane (DABCO, 99%, Aldrich), diethylene glycol (DEG, 98%, Aldrich) and synthetic hydroxyapatite (HA, Riedle-de Haën, Poznań, Poland) were used without further purification. ε-Caprolactone (2-oxepanone, 99%, CL, Aldrich) was dried and distilled over CaH_2_ at reduced pressure before use.

### 3.2. Synthesis of Poly(ε-caprolactone) Diols

The CL, DEG and CA were weighed (under dry argon) into a cylindrical glass reactor. Before the reactions, the monomer, glycol and enzyme were dried *in vacuo* at room temperature for 1 h. The reaction vessel was placed into an oil bath. Polymerization of the CL (0.05 mol) in the presence of DEG (0.00125–0.0025 mol) and 100 mg CA was carried out in bulk (under dry argon at 70 °C for 14 days). After the polyreaction time was complete, the mixture was dissolved in CH_2_Cl_2_ and the insoluble enzyme was removed by filtration. Next, the obtained solution was washed with cold MeOH using vigorous stirring. The operation was repeated three times [31]. The final products (poly(ε-caprolactone) diols, PCL diols) were dried *in vacuo* at room temperature for 48 h.

#### FTIR and NMR Data

^1^H NMR (CDCl_3_, δ, ppm): 4.03 [2H, t, –CH_2_CH_2_CH_2_CH_2_C**H**_2_OC(O)–], 2.27 [2H, t, –CH_2_CH_2_CH_2_CH_2_C**H**_2_COO–], 1.61 [4H, m, –CH_2_C**H**_2_CH_2_C**H**_2_CH_2_COO–], 1.36 [2H, m, –CH_2_CH_2_C**H**_2_CH_2_CH_2_COO–];

^13^C NMR (CDCl_3_, δ, ppm): 173.3 [–**C**(O)O–], 63.9 [–CH_2_CH_2_CH_2_CH_2_**C**H_2_OC(O)–], 33.8 [–CH_2_CH_2_CH_2_CH_2_**C**H_2_COO–], 28.1 [–CH_2_CH_2_CH_2_**C**H_2_CH_2_OC(O)–], 25.4 [–CH_2_CH_2_CH_2_**C**H_2_CH_2_COO–], 24.4 [–CH_2_CH_2_**C**H_2_CH_2_CH_2_COO–];

FTIR (KBr, cm^−1^): 2950 (υ_as_CH_2_), 2865 (υ_s_CH_2_), 1730 (υC=O), 1240 (υ_as_COC), 1190 (υOC–O), 1170 (υ_s_COC).

### 3.3. Synthesis of Polyurethanes

The PUs were prepared following a two-step, pre-polymer synthesis method. The isocyanate index was about 1.05. First, all the reactants (HMDI, PEAD diol, PCL diol, BD) were dried *in vacuo* for 1 h at 60 °C. The reactor was vacuumed and then purged with argon. The polyols and catalyst (DABCO) were first mixed at 80–100 °C in a three-necked flask equipped with a stirrer and thermometer. Next, HMDI was added to the reaction mixture and all the components were mixed vigorously for about 5 min. The temperature of the reactor was reduced to 70–80 °C. A chain extender (BD) was then slowly added to the reaction mixture. The reaction was kept at 70–80 °C for 3 h. Next, the product was conditioned *in vacuo* at 50 °C for 24 h. The synthesized PUs were dissolved in DMSO and precipitated into distilled water. Next, precipitated PUs were dried *in vacuo* at 40–50 °C for one week.

#### FTIR and NMR Data

^1^H NMR (DMSO-d_6_, δ, ppm): 6.87 [1H, s, –OC(O)N**H**–], 4.33 [4H, t, –NHCOO–C**H**_2_CH_2_CH_2_C**H**_2_OC(O)NH–], 3.91 [2H, t, –NHC(O)OC**H**_2_–], 3.78–3.65 [4H, m, –NHCOO–CH_2_C**H**_2_C**H**_2_CH_2_OC(O)NH– and 4H, t, –C(O)OC**H**_2_C**H**_2_O–], 3.17 [2H, t, –C**H**_2_NHC(O)O–], 2.30–2.25 [2H, t, CH_2_C**H**_2_COO–], 1.60–1.50 [2H, m, –C**H**_2_CH_2_NHC(O)O– and 4H, m, –CH_2_C**H**_2_CH_2_C**H**_2_CH_2_COO–], 1.40–1.30 [2H, m, –C**H**_2_CH_2_CH_2_NHC(O)O– and 2H, m, –CH_2_CH_2_C**H**_2_CH_2_CH_2_COO–];

^13^C NMR (DMSO-d_6_, δ, ppm): 173.1 [–**C**(O)O–], 156.1 [–NH**C**(O)O–], 63.3 [–OC(O)**C**H_2_–], 33.8 [–CH_2_CH_2_CH_2_CH_2_**C**H_2_COO–], 29.2 [–**C**H_2_NHC(O)O–], 28.5 [–NHC(O)NH**C**H_2_–], 24.9–25.9 [–**C**H_2_–];

FTIR (KBr, cm^−1^): 3323 (υN–H), 2938 (υ_as_CH_2_), 2859 (υ_s_CH_2_), 1734 (υC=O, polyester), 1623 (υC=O, ester), 1535 (δN–H, urethane).

### 3.4. Cytotoxicity Assays

The luminescent bacteria *V. fischeri* and two ciliated protozoans *S. ambiguum* and *T. termophila* were used to evaluate the cytotoxicity of the obtained PCL diols and PUs. The cytotoxicity tests were carried out according to procedures described in our earlier papers [32,33].

### 3.5. Composite Production

The previously synthesized PUs were first dissolved in DMSO at a concentration of 10%–20% (*w*/*v*). Next, the PUs solution were mixed with HA. Pores were created by mixing the mixture of PUs and HA with 0.5 g of NaCl crystals per 1.5 g of PU. The PU/salt mixtures were poured into a mould. Next, the mould were dried *in vacuo* at 40–50 °C for 24–48 h. The samples were washed for 24 h in distilled water to remove NaCl. The composite samples were later dried *in vacuo* at room temperature for about one week.

### 3.6. Clodronate Impregnation of the Polyurethane Composites

CLO was incorporated into the PU composites by immersing the material into an aqueous drug solution of known concentration. The solution was pulled into the pores of the biomaterials by repeated five-cycles of vacuum/argon. PU composites were dried *in vacuo* at room temperature until the weight of the impregnated materials remained unchanged. The gain in weight of the PU composites following impregnation was taken as the weight of the CLO incorporated into the biomaterials.

### 3.7. Clodronate Release from the Composites

The composite BPDDS were incubated in a phosphate buffer solution (PBS) (pH 7.4) at a ratio of 15 mg of composite to 1 mL of buffer at 37 °C. The mixture was stirred under constant agitation (50 cycles/min) and a sample was removed at selected intervals followed by fresh buffer replacement. The quantity of the released CLO was determined from the calibration curve previously obtained under the same conditions and analysed by means of the high performance liquid chromatography with charged aerosol detector (HPLC CAD) method.

### 3.8. Degradation Test

The hydrolytic degradation of the PUs and PU composites was measured by immersion for eight weeks in a PBS at 37 °C. After a certain period, the biomaterials were completely dried in a vacuum oven at 35 °C. Three individual experiments were performed in the degradation test, and then the average value was calculated. The degree of degradation was determined from the weight loss (*W_L_*) of the polymeric samples according to the equation: *W_L_* = [(*W*_0_ − *W*_d_)/*W*_0_] × 100 (%), where *W*_0_ is the initial weight of the polymer sample and *W*_d_ is the weight of the dry polymer sample after degradation.

### 3.9. Measurements

^1^H and ^13^C NMR spectra of the PCL diols and PUs were recorded on a Varian 300 MHz spectrometer using CDCl_3_ or DMSO-d_6_ as a solvent. Tetramethylsilane was served as the internal standard. The FTIR spectra were measured from KBr pellets (PerkinElmer spectrometer, PerkinElmer, Warsaw, Poland).

The molar mass and molar mass distributions of the PCL diols were determined using a GPC instrument (GPC Max + TDA 305, Viscotek, Malvern, UK) equipped with Jordi DVB Mixed Bed columns (one guard and two analytical, Viscotek) at 30 °C in CH_2_Cl_2_ (HPLC grade, Sigma-Aldrich, Poznań, Poland) at a flow rate of 1 mL/min with RI detection and calibration based on narrow PS standards (ReadyCal Set, Fluka). The results were processed using the OmniSEC software (ver. 4.7) (Viscotek).

The MALDI-TOF MS spectra were performed in linear mode on an ultrafleXtreme™ (Bruker Daltonics, Poznań, Poland) mass spectrometer using a nitrogen gas laser and HABA as a matrix. The polymer samples were dissolved in tetrahydrofuran (THF) (5 mg/mL) and mixed with a solution of HABA.

Polymer viscosity was measured in DMF (at 30 °C) on a Stabinger Viscometer SVM 3000 (Anton Paar’s, Graz, Austria). The concentrations of the PU solutions in DMF were 0.2%, 0.4%, 0.6%, 0.8% and 1%. The viscosity-average molecular weight was calculated with the Mark-Houwink equation using the following constants: K = 6.80 × 10^−5^ dL/g and α = 0.86 [34].

The surface morphologies were studied by scanning electron microscope (SEM, LEO 435VP (Zeiss, Jena, Germany)) so as to compare them with the initial morphologies.

The fail stress (*F_S_*), stress at 100% elongation (*S_100_*), elongation at break (ε) and Shore’a hardness (*Sh_H_*) of the PU samples were measured using a Zwick model 1445 tester. *F_S_*, *S_100_* and ε were determined according to national standard PN-ISO 37:2007. *Sh_H_* was determined according to national standard PN-80/C-04238 [35].

The density and porosity values of the PU composites were measured by the liquid displacement method [36]. Ethanol (EtOH) was used as the displacement liquid. A dry composite sample was placed in a cylinder filled with a predetermined volume of EtOH (*V*_1_). Next, the cylinder was placed *in vacuo* for 20 min. The total volume of EtOH containing the composite sample was recorded as *V*_2_ and the residual EtOH volume was recorded as *V*_3_. Three individual measurements were performed and then the average value was calculated.

The amount of open pores in the PU composites (*P*) was calculated according to the following equation:

*P* (%) = (*V*_1_ − *V*_3_)/(*V*_2_ − *V*_3_) × 100%

where (*V*_2_ − *V*_3_) denotes the total volume of the composite sample and (*V*_1_ − *V*_3_) denotes the volume of ethanol retained in the composite sample.

The density of the composite (*d*) was expressed as:

*d* = *W*/(*V*_2_ − *V*_3_)

where *W* refers to the weight of the sample.

The quantity of the released CLO was analysed by means of HPLC CAD using the UHPLC Dionex Ultimate 3000 analytical system with a CAD detector. Chromatographic separations were carried out using the Luna C8 column (250 × 4.6 mm, 5 μm). The calibration curve was obtained by the analysis of different concentrations of CLO in PBS solutions (0.05–2.00 mg/mL). The analytical method was validated by the Pharmaceutical Research Institute in Poland.

## 4. Conclusions

New porous composite bisphosphonate delivery systems were prepared from biodegradable polyurethanes and nanocrystalline hydroxyapatite. The obtained polymer matrices were non-toxic. The rates of clodronate release were shown to be directly dependent upon the nature of the obtained polyurethanes and the porosity of the composites. The results demonstrate that the polyurethane/hydroxyapatite composites are promising materials for the controlled release of clodronate and they can find practical applications as effective medium- or long-term implantation drug delivery systems.
